# Developmental regulation of lupulin gland-associated genes in aromatic and bitter hops (*Humulus lupulus* L.)

**DOI:** 10.1186/s12870-021-03292-z

**Published:** 2021-11-13

**Authors:** Josef Patzak, Alena Henychová, Jaroslav Matoušek

**Affiliations:** 1 Hop Research Institute Co., Ltd., Kadaňská 2525, 438 01 Žatec, Czech Republic; 2 Biology Centre ASCR v.v.i, Department of Molecular Genetics, Institute of Plant Molecular Biology, Branišovská 31, 37005 České Budějovice, Czech Republic

**Keywords:** Hop, *Humulus lupulus*, Bitter acids, Glandular trichome development, Lupulin gland, Differential gene expression

## Abstract

**Background:**

Hop (*Humulus lupulus* L.) bitter acids are valuable metabolites for the brewing industry. They are biosynthesized and accumulate in glandular trichomes of the female inflorescence (hop cone). The content of alpha bitter acids, such as humulones, in hop cones can differentiate aromatic from bitter hop cultivars. These contents are subject to genetic and environmental control but significantly correlate with the number and size of glandular trichomes (lupulin glands).

**Results:**

We evaluated the expression levels of 37 genes involved in bitter acid biosynthesis and morphological and developmental differentiation of glandular trichomes to identify key regulatory factors involved in bitter acid content differences. For bitter acid biosynthesis genes, upregulation of humulone synthase genes, which are important for the biosynthesis of alpha bitter acids in lupulin glands, could explain the higher accumulation of alpha bitter acids in bitter hops. Several transcription factors, including HlETC1, HlMYB61 and HlMYB5 from the MYB family, as well as HlGLABRA2, HlCYCB2–4, HlZFP8 and HlYABBY1, were also more highly expressed in the bitter hop cultivars; therefore, these factors may be important for the higher density of lupulin glands also seen in the bitter hop cultivars.

**Conclusions:**

Gene expression analyses enabled us to investigate the differences between aromatic and bitter hops. This study confirmed that the bitter acid content in glandular trichomes (lupulin glands) is dependent on the last step of alpha bitter acid biosynthesis and glandular trichome density.

**Supplementary Information:**

The online version contains supplementary material available at 10.1186/s12870-021-03292-z.

## Background

Hop (*Humulus lupulus* L.) is a diploid, dioecious, perennial climbing plant belonging to the *Cannabaceae* family. Female plants are cultivated for the commercial production of inflorescences (cones), which are mainly used in the brewing industry but also commonly used in the production of pharmaceuticals and cosmetics [1]. In cones, flavour-active secondary metabolites are biosynthesized and accumulate in glandular trichomes and lupulin glands. There are three key classes of natural products: alpha and beta bitter acids (humulone and lupulone derivatives, respectively), prenylated flavonoids (primarily xanthohumol and desmethylxanthohumol) and essential oils composed mainly of myrcene, α-humulene and β-caryophyllene [2].

The contents of alpha and beta bitter acids in hop cones (5 to 30% of the dry weight) are major chemical characteristics and economical traits of different cultivars during hop production. Their biosynthesis requires precursor sources that directly originate from primary sucrose metabolism and amino acid precursors such as leucine, isoleucine, valine and phenylalanine [3]. Phenylalanine is biosynthesized by the shikimate pathway and others by the branched-chain amino acid (BCAA) pathway in chloroplasts. The final step in the BCAA pathway is catalysed by the enzyme branched-chain amino transferase 2 (HlBCAT2). For bitter acid biosynthesis, BCAAs are then degraded in the mitochondria by branched-chain amino transferase 1 (HlBCAT1) and branched-chain keto-acid dehydrogenase (HlBCKDH) to isovaleric, 2-methylbutyric and isobutyric acids, respectively [4]. Coenzyme A is ligated to these precursors by two branched short-chain fatty acid CoA ligases (HlCCL2 and HlCCL4) in the cytosol [5]. Valerophenone synthase (HlVPS) synthesizes phloroisovalerophenone (PIVP), phloro-2-methylbutyrophenone (PMBP) and phloroisobutyrophenone (PIBP) from precursors and three malonyl-CoAs from phenylalanine [6].

Isoprenoids are a large and highly diverse group synthesized from common prenyl diphosphate precursors. Plants synthesize isopentenyl diphosphate (IPP) via mevalonate and methylerythritol phosphate (MEP) pathways [7]. The MEP pathway is more efficient in hop lupulin glands according to transcriptome [4] and proteome [3] studies. Terpenic compounds are the main components of hop essential oils.

Bitter acids and prenylflavonoids are additional specific compounds of prenylation in lupulin glands. Both are prenylated by a functional heterocomplex of two aromatic prenyltransferases, HlPT1L and HlPT2, in chloroplasts [8–10]. HlPT1L catalyses the first prenylation step, and HlPT2 catalyses the next two prenylation steps. The first step produces alpha bitter acid precursors, such as deoxyhumulone, deoxyadhumulone and deoxycohumulone, and the second step produces beta bitter acids, such as lupulone, adlupulone and colupulone. Alpha bitter acids (humulone, adhumulone and cohumulone) are finally produced by humulone synthase (HlHS1 and HlHS2) via the oxygenation of deoxy-precursors [3]. The content of individual analogues of alpha and beta bitter acids is determined by the ratios of precursors and is genetically imprinted in the genomes of individual genotypes; for example, cohumulone (from 12 to 55% of alpha acids) and colupulone (from 30 to 80% of beta acids) contents are very stable and heritable traits [2].

Since the expression of biosynthetic genes is the highest in lupulin glands, there is a tissue-specific regulatory network of transcription factors mainly from the bHLH, MYB, WDR and WRKY families [1]. Promoters of bitter acid and prenylflavonoid biosynthesis pathway genes are regulated by heterotrimeric ternary MBW (HlMyb3/HlbHLH2/HlWDR1 or HlMYB2/HlbHLH2/HlWDR1) complexes [11], binary (HlbHLH2/HlWDR1 or HlWRKY1/HlWDR1) transcription activation complexes [11, 12] or individual transcription factors (HlWRKY1 or HlMyb8) [13, 14]. This regulation allows hop plants to react to developmental changes, environmental stresses and weather conditions, which influence bitter acid contents.

The bitter acid content in hop cones varies across hop cultivars due to their genetic background. Hop cultivars are divided into aromatic hops, with low alpha bitter acid contents in dry cones ranging from 0.5 to 8%, and bitter hops, with alpha bitter acid contents in dry cones over 9 to 23% [15]. It was found that the content of alpha bitter acids very strongly and significantly correlated with the number and size of lupulin glands in hop cones [2, 16, 17]. Therefore, bitter acid contents can be increased either by increasing their production inside secretory cells or by increasing the density of glands on individual cones. The development of multicellular glandular trichomes includes the enlargement of single epidermal cells, followed by several polarized and localized cell divisions and remodelling to generate branched or unbranched structures perpendicular to the epidermal surface. The lupulin glands develop on the abaxial surface of the leaf, bract or bracteole primordia and continue to form until expansion stops [1]. There are already molecular data for genes that play a specific role in glandular trichome development, especially transcription factors, cell cycle regulators, and receptors involved in phytohormone-induced signalling cascades [18].

The model plant *A. thaliana* is the most utilized for the developmental study of trichomes, which are single-celled and non-glandular [19]. The current model includes transcription factors that function as positive or negative regulators of trichome formation, upstream regulators of these two groups of regulators, and downstream components [20]. The positive regulators belong to three protein classes and include a WD40-repeat protein TRANSPARENT TESTA GLABRA1 (TTG1), three R2R3 MYB-related transcription factors GLABRA1 (GL1, MYB23, MYB5) and four basic helix-loop-helix (bHLH)-like transcription factors GLABRA3 (GL3), ENHANCER OF GLABRA3 (EGL3), TRANSPARENT TESTA (TT8), and MYC-1. They act partially redundantly and form a multimeric activator complex, also known as the MYB-bHLH-WD40 (MBW) complex, which binds the promoter of GLABRA2 (GL2) and several homeodomain (HD-ZIP class IV) transcription factors [19]. GL2 encodes a homeodomain protein required for subsequent phases of trichome morphogenesis, such as endoreduplication, branching, and maturation of the cell wall. The MBW activator complex is usually negatively regulated by single-repeat R3 MYBs, such as CAPRICE (CPC), TRIPTYCHON (TRY), ENHANCER OF TRY and CPC1 (ETC1), ETC2, TRICHOMELESS1 (TCL1) and TCL2/CPL4 [20]. Additionally, several C2H2 zinc finger protein transcription factor genes, including GLABROUS INFLORESCENCE STEMS (GIS, GIS2) and ZINC FINGER PROTEIN 8 and 5 (ZFP8, ZFP5), controlling GL1 and GL3 of the core MBW complex, have also been identified [19, 20].

In contrast, knowledge of the development of glandular trichomes is very limited, and some data have shows that glandular trichome pathways are not as conserved as the nonglandular trichome pathways known in *Arabidopsis*. What we know about glandular trichome development is mostly from work on *Artemisia*, tomato and tobacco [19].

In tomato, several genes have been reported that are required for the proper development and function of different types of glandular trichomes. The initiation and development of type I trichomes was controlled by a woolly (Wo) gene encoding a class IV homeodomain-leucine zipper protein homologue of *Arabidopsis* GL2 and a B-type cyclin gene, SlCycB2 (possibly regulated by Wo). A hairy phenotype was caused by the overproduction of mutant alleles of Wo in type I trichomes. However, the suppression of Wo or SlCycB2 expression by RNA interference decreased trichome density in tomato [21, 22]. Addititonally, three transcription factors, Woolly, Hair, and Mixta-like, were found to be necessary for the development of type I glandular trichomes, while Woolly, Hair, and Myc1 were found to be required for type VI glandular trichome development [19, 23]. SlMYC1 is also involved in the transcriptional regulation of several terpene synthases [24]. Trichome formation is also inhibited by a tomato TRY orthologue. Therefore, some of the MYB-bHLH-WD40 complex elements might be conserved in tomato together with HD-ZIP and C2H2 factors [19].

A recent RNA-sequencing analysis showed that orthologues of *Arabidopsis* genes involved in trichome formation via the MYB-bHLH-WD40 regulatory mechanism were expressed in tobacco trichomes. Nevertheless, the overexpression of a Wo transgene had no influence on their transcription and trichome induction. In contrast, Wo and SlCycB2 homologues involved in trichome formation in asterids were significantly upregulated in Wo transgenic tobacco plants [18, 25]. The expression of a C2H2-zinc-finger transcription factor, NbGIS, or the tomato Wo gene both led to more glandular trichomes being produced [26].

In *Artemisia annua*, AaMYB1 overexpression induces a higher density of trichomes [20]; AaHD1, a homeodomain-leucine zipper TF, is necessary for JA-mediated glandular trichome initiation [18]; and the AaHD8/AaMIXTA1 complex regulates trichome initiation and cuticle development [27]. Increased expression of AmMIXTA from *Antirrhinum majus* also leads to longer glandular trichomes in tobacco leaves [20]. Additionally, some other positive regulators of glandular trichome formation have been found, such as GoPGF, a bHLH TF from cotton, and three HD-Zip TFs, CsGL1, CsGL2 and CsGL3, in cucumber [1, 18]. Collectively, results from these species indicate that similar types of transcription factors control glandular trichome initiation and development [19].

In this study, we evaluated the expression levels of 37 genes involved in bitter acid biosynthesis and the morphological and developmental differentiation of lupulin glands in aromatic and bitter hops to identify key regulatory factors for bitter acid content differences.

## Results

### Genes involved in bitter acid biosynthesis

**Fig. 1:**
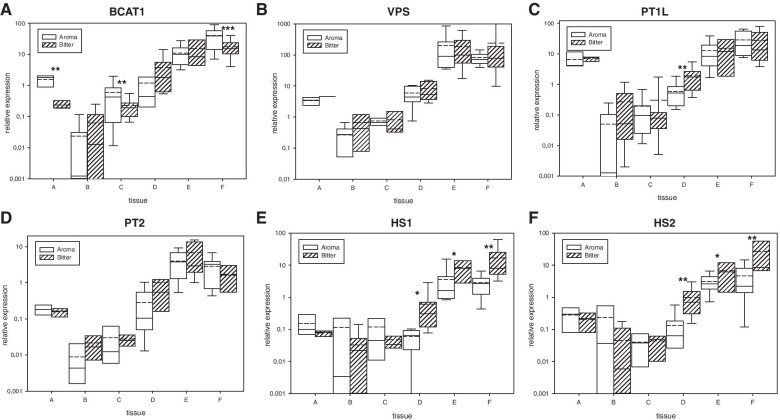
The relative expression of bitter acid biosynthesis genes **A**: HlBCAT1, **B**: HlVPS, **C**: HlPT1L, **D**: HlPT2, **E**: HlHS1 and **F**: HlHS2 (see Table 1) relative to reference genes in different tissues of aromatic and bitter hop cultivars. Tissues: A – apex, B – leaf, C – bract without lupulin, D – flower, E – young cone, and F – lupulin gland. Significant t-test group differences at the following probability levels: * - *P* < 0,1, ** - *P* < 0,05, and *** - *P* < 0,01. straight line - median, dashed line – average, and box – 95% percentile ± standard deviation

Glandular trichomes and lupulin glands are generally dedicated to the synthesis of specialized hop secondary metabolites. Bitter acids are major chemical compounds with economic impacts on hop production. Therefore, we evaluated expression differences of known bitter acid biosynthetic pathway enzyme genes between aromatic and bitter hop cultivars. We found that the gene expression of the HlBCAT1, HlVPS, HlPT1L and HlPT2 genes (Fig. 1A-D) was upregulated in lupulin glands (from 170 to 1000 times compared to leaves) and downregulated in leaves and bracts. There were no significant differences in these genes between aromatic and bitter hop cultivars, except for HlBCAT1 and HlPT1L. Two monooxygenase 2 genes, HlHS1 and HlHS2, were 5.8 times more highly expressed in the lupulin glands of bitter hop cultivars than in those of aromatic hop cultivars (Fig. 1E and F). This trend was also evident in flower and young cone samples.

### Transcription factors involved in bitter acid biosynthesis pathways

**Fig. 2:**
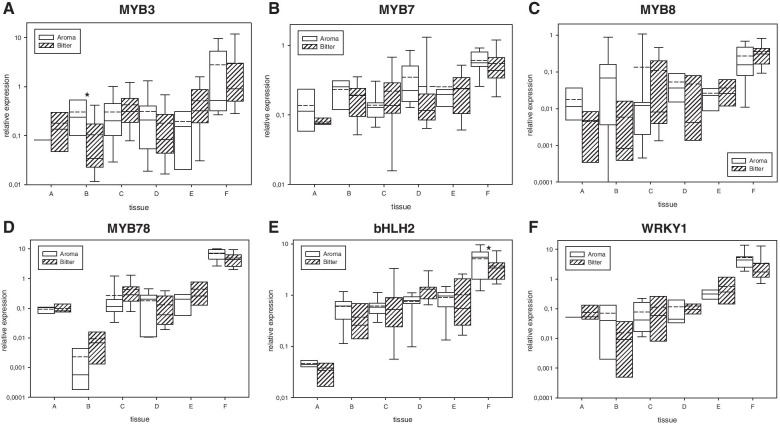
The relative expression of bitter acid biosynthesis transcription factors **A**: HlMYB3, **B**: HlMYB7, **C**: HlMYB8, **D**: HlMYB78, **E**: HlbHLH2 and **F**: HlWRKY1 relative to reference genes (see Table 1) in different hop tissues. Tissues: A – apex, B – leaf, C – bract without lupulin, D – flower, E – young cone, and F – lupulin gland. Significant t-test group differences at the following probability levels: * - *P* < 0,1, ** - *P* < 0,05, and *** - *P* < 0,01. straight line - median, dashed line – average, and box – 95% percentile ± standard deviation

Relative expression differences of biosynthesis pathway genes can be caused by the regulatory networks of involved transcription factors. All of the studied transcription factors, which had previously been suggested to regulate bitter acid biosynthesis, were highly expressed in lupulin glands (Fig. 2A-F). Upregulation was not as evident for the HlMYB3, HlMYB7 and HlMYB8 transcription factors as for HlMYB78, HlbHLH2 and HlWRKY1. HlMYB78 was essentially downregulated in leaf tissue (Fig. 2D) and HlbHLH2 in apex tissue (Fig. 2E). There were no significant differences between aromatic and bitter hop cultivars, except for HlMYB3 in leaves (Fig. 2A) and HlbHLH2 in lupulin glands (Fig. 2E).

### Transcription factors involved in trichome initiation and development

The alpha bitter acid content is dependent on lupulin gland density; therefore, the regulation of glandular trichome initiation and development is important for breeding and hop cultivar improvement. Knowledge of the trichome developmental regulation network is limited in hop; therefore, we tried to find key regulatory factors for relative differential expression within aromatic and bitter hop cultivars.

**Fig. 3:**
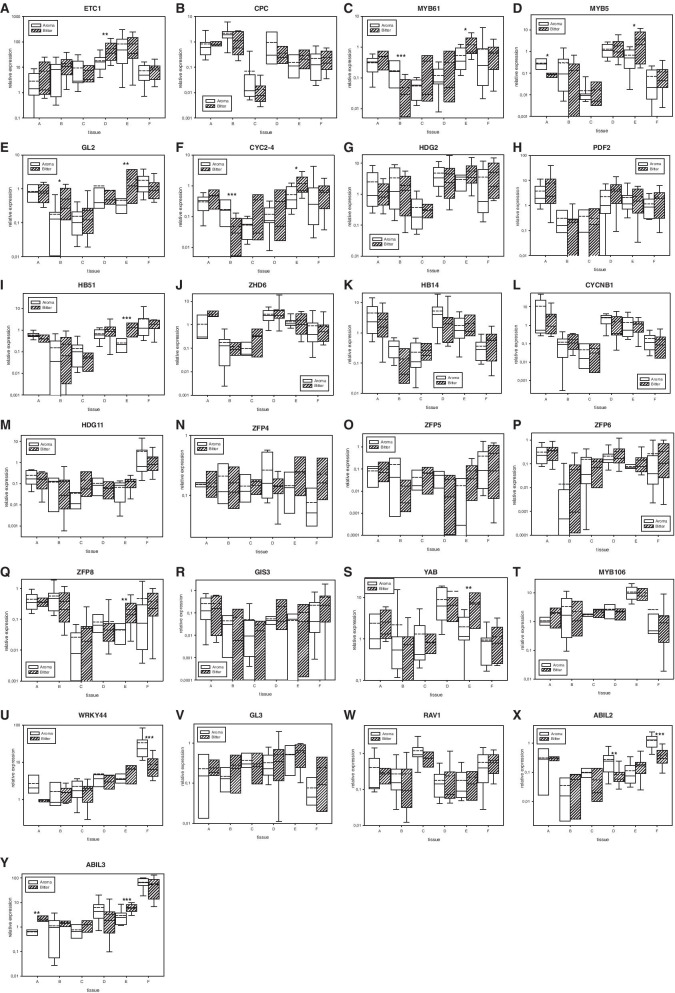
The relative expression of reference genes for trichome-regulating transcription factors **A**: HlETC1, **B**: HlCPC, **C**: HlMYB61, **D**: HlMYB5, **E**: HlGL2, **F**: HlCYCB2–4, **G**: HlHDG2, **H**: HlPDF2, **I**: HlHB51, **J**: HlZHD6, **K**: HlHB14, **L**: HlCYCNB1, **M**: HlHDG11, **N**: HlZFP4, **O**: HlZFP5, **P**: HlZFP6 **Q**: HlZFP8, **R**: HlGIS3, **S**: HlYABBY1, **T**: HlMYB106, **U**: HlWRKY44, **V**: HlGL3, W: HlRAV1, **X**: HlABIL2 and **Y**: HlABIL3 (see Table 1) in different tissues of aromatic and bitter hop cultivars. Tissues: A – apex, B – leaf, C – bract without lupulin, D – flower, E – young cone, and F – lupulin gland. Significant t-test group differences at the following probability levels: * - *P* < 0,1, ** - *P* < 0,05, and *** - *P* < 0,01. straight line - median, dashed line – average, and box – 95% percentile ± standard deviation

We found that the negative regulator of trichome development HlETC1 was 3 times more highly expressed in flowers of bitter hop cultivars than in those of aromatic hop cultivars (Fig. 3A). However, we did not find any differences for similar HlCPC negative regulators, and their expression profiles were different (Fig. 3B). Two other MYB-related transcription factors, HlMYB61 and HlMYB5, were both 2.2 times more highly expressed in young cones of bitter hop cultivars than in those of aromatic hop cultivars (Fig. 3C and D). Differential expression was also found in leaves for HlMYB61 (Fig. 3C) and apexes for HlMYB5 (Fig. 3D).

For homeobox-leucine zipper proteins, HlGLABRA2 was 4.2 times more highly expressed in young cones and 2.8 times more highly expressed in leaves of bitter hop cultivars than in those of aromatic hop cultivars (Fig. 3E). The relative expression of the HlCYCB2–4 homologue was 3 times higher in young cones of bitter hop cultivars than in those of aromatic hop cultivars (Fig. 3F). However, the Wo gene homologues HlHDG2 and HlPDF2 were not differentially expressed in any tissue (Fig. 3G and H). HlHB51 was 5 times more highly expressed in young cones of bitter hop cultivars than in those of aromatic hop cultivars (Fig. 3I). HlZHD6, HlHB14 and HlCYCNB1 had similar expression profiles across hop tissues (Fig. 3J, K and L). HlHDG11 was upregulated in lupulin glands (Fig. 3M).

Of the five studied C2H2 zinc finger proteins (Fig. 3N-R), HlZFP8 was 4.6 times more highly expressed in young cones of bitter hop cultivars than in those of aromatic hop cultivars (Fig. 3P). HlZFP4 (Fig. 3N) and HlZFP5 (Fig. 3O) were also nonsignificantly upregulated in young cones.

Regarding other regulatory factors, the axial regulator HlYABBY 1 was 3.7 times more highly expressed in young cones of bitter hop cultivars than in those of aromatic hop cultivars (Fig. 3S). The MIXTA homologue HlMYB106 was upregulated in young cones compared to other tissues with no differences between aromatic and bitter hop cultivars (Fig. 3T). The relative expression of the HlWRKY44 factor was 3.8 times higher in the lupulin glands of aromatic hop cultivars than in those of bitter hop cultivars (Fig. 3U). There were no significant differences in the bHLH factor HlGL3 (Fig. 3V) and AP2/ERF and B3 domain-containing transcription factor HlRAV1 (Fig. 3W) between aromatic and bitter hop cultivars. For Abl interactor-like proteins, HlABIL2 was 3.3 and 3.2 times more highly expressed in flowers and lupulin glands of aromatic hop cultivars than in those of bitter hop cultivars (Fig. 3X). However, HlABIL3 was 3.4 and 2.1 times more highly expressed in the apexes and young cones of bitter hop cultivars than in those of aromatic hop cultivars (Fig. 3Y).

## Discussion

### Genes involved in bitter acid biosynthesis

Today, all bitter acid biosynthetic pathway enzyme genes [3–10] that are predominantly and highly expressed in lupulin glands are known. Expression differences between aromatic and bitter hop cultivars have not yet been studied. We confirmed that bitter acid biosynthetic genes are upregulated in lupulin glands and downregulated in leaves and bracts. Significant differences between aromatic and bitter hop cultivars were found for the HlBCAT1 and HlPT1L genes. HlBCAT1 is localized to the mitochondria and is involved in amino acid biosynthesis [4]. Upregulation of this gene in the apex, bract and lupulin gland of aromatic cultivars is connected with the amino acid composition. HlPT1L is also involved in prenylflavonoid biosynthesis [8, 9], and upregulation of this gene in flowers of bitter cultivars is connected with higher levels of these compounds. Recently, two monooxygenase 2 genes [3] (HlHS1 and HlHS2) required for the last step of alpha bitter acid biosynthesis were found to be upregulated in the lupulin glands, flowers and young cones of bitter hop cultivars. From these findings, it can be supposed that HlHS genes are important for the alpha bitter acid content in hop cones together with lupulin gland density [2, 16, 17].

### Transcription factors involved in bitter acid biosynthesis pathways

Bitter acid biosynthesis pathway genes are regulated by transcription factor networks [11–14]. We studied the relative expression of six known transcription factors and confirmed their upregulation in lupulin glands. Two factors, HlMYB3 and HlbHLH2, were differentially expressed in aromatic and bitter hop cultivars in the leaves or lupulin glands, respectively. Both genes are involved in prenylflavonoid biosynthesis [11–14], and differences in this pathway are connected to these compounds. Differences in HS gene expression have not been found to be related to any of the studied transcription factors. It is likely that either other transcription factors or changes in cis-acting regulatory DNA elements on promoter sequences can produce this upregulation. A more detailed study will be necessary in the future.

### Transcription factors involved in trichome initiation and development

Because lupulin gland density is correlated with alpha bitter acid content [2, 16, 17], we studied 25 candidate regulatory factors and genes involved in glandular trichome initiation and development.

One of the essential regulators responsible for glandular trichome density formation [19, 20], the negative regulator of trichome development HlETC1, was upregulated in flowers of bitter hop cultivars. Its expression was downregulated in lupulin glands, similar to the results found in a previous study [1]. A similar negative regulator of HlCPC was differentially expressed in hop tissues. The amino acid (AA) sequence of HlETC1 has 55.7% homology to *Arabidopsis thaliana At*CPC, 54.2% homology to *At*ETC3, 48% homology to *At*ETC1 and 43.2% homology to *At*ETC2. Although the AA homology between HlETC1 and HlCPC was only 43.6%, the homology of CPC to *A. thaliana* genes was similar to that of ETC1 (61.8% to *At*CPC, 61.5% to *At*ETC2, 56.9% to *At*ETC1 and 44.7% to *At*ETC3). Therefore, we cannot determine the exact role of this HlETC1 gene in controlling trichome formation.

Other MYB-related transcription factors, such as HlMYB61 and HlMYB5, were also upregulated in young cones of bitter hop cultivars. They are act as GLABRA1 transcription factors in the regulation of mucilage synthesis, seed coat development and trichome morphogenesis in *A. thaliana* [28] or as AaMYB1 transcription factors in the regulation of terpene synthesis and trichome development in *Artemisia annua* [29]. Their pleiotropic function was identified based on their differential expression in leaf and apex tissues. These genes may be part of generally conserved MYB-bHLH-WD40 complex regulation, which is involved in lupulin gland development. This result was confirmed by the upregulation of HlGLABRA2 in young cones and leaves of bitter hop cultivars. The GL2 gene possibly regulates the expression of SlCycB2 during the initiation and development of type I trichomes in tomato [21, 22], and its homologue HlCYCB2–4 was also upregulated in young cones of bitter hop cultivars.

The previously identified lupulin-upregulated homeobox-leucine zipper protein HlHB51 was also upregulated in young cones of bitter hop cultivars [1]. ATHB-51 interacts with the meristem regulator LEAFY and participates in trichome formation, leaf morphogenesis and floral meristem determinacy in *Arabidopsis* [30], and it is an essential regulator of multicellular trichome development in *Cucumis sativum* [31]. Similarly expressed homeobox-leucine zipper proteins HlZHD6, HlHB14 and HlCYCNB1 probably participate in meristem determinacy and development. HlHDG11 was similarly upregulated in lupulin glands in a previous study [1]. This gene is involved in epidermal cell differentiation, and HDG11 mutants show excess branching of the trichome in *A. thaliana* [32].

In a previous study, the axial regulator HlYABBY1 was significantly downregulated in lupulin glands and young cones [1], as in our results. We can suppose from its upregulation in young cones of bitter hop cultivars that this gene may be involved in lupulin gland development at abaxial sites of bracts and bracteoles [33].

The MIXTA transcription factor is also considered a positive regulator of glandular trichome formation [19, 20]. It was obvious from our results for its homologue HlMYB106 that this gene is a general transcription factor for glandular trichome development.

HlWRKY44, TRANSPARENT TESTA GLABRA2 (TTG2) factor, regulates trichome-specific TTG1, GL2, GL3 and TRY factors [34]. Its downregulation in the lupulin glands of bitter hop cultivars probably results in downregulation of the MYB-related transcription factor TRY, a negative regulator of trichome development. However, WRKY44 is also involved in the biosynthesis of proanthocyanins in fruits [35] or mucilage and tannins in seed coats [36].

The homologue of the bHLH factor HlGL3 was downregulated in lupulin glands without differences between aromatic and bitter hop cultivars. Therefore, we cannot confirm that this gene is a part of the MYB-bHLH-WD40 complex, and thorough combinatorial analysis will be necessary [11].

Expression differences in hop cone tissues were previously found [1] for AP2/ERF and the B3 domain-containing transcription factor HlRAV1, which were confirmed despite a lack of influence on glandular trichome density or alpha bitter acid content. Other genes that were overexpressed in lupulin glands include the Abl interactor-like proteins HlABIL2 or HlABIL3 [1], which are involved in the regulation of actin and microtubule organization as part of a SCAR/WAVE complex that activates the ARP2/3 complex [37]. Based on the expression differences in apexes, flowers, young cones or lupulin glands, we cannot exactly determine what aspect of trichome, cone or seed development is influenced, even if these genes play a role in the development of plant cell shape and can cause a distortion in the trichome phenotype [38].

## Conclusions

Gene expression analyses enabled us to identify differences between aromatic and bitter hops. This study confirmed that the bitter acid content in glandular trichomes (lupulin glands) is dependent on the last step of alpha bitter acid biosynthesis and glandular trichome density. Humulone synthase genes (two analogues) are important for alpha bitter acid content in the glandular trichomes of hop cones. Differential gene expression analyses showed that the MYB transcription factors HlETC1, HlMYB61 and HlMYB5, homeobox-leucine zipper protein HlGLABRA 2, C2H2 zinc finger protein HlZFP8 and axial regulator HlYABBY1 may be key regulatory factors for lupulin gland density and morphological and developmental differentiation of glandular trichomes inside hop cones. Analyses also showed that glandular trichome initiation and development are controlled by a large regulatory network and should be studied in detail in the future.

## Methods

### Plant materials

The hop plants used were grown under standard agronomic conditions in experimental fields on the Steknik farm of the Hop Research Institute in Zatec (Saaz), CR. Based on general long-term knowledge, we selected the “aromatic” hop cultivars Saaz, Fuggle, Hallertauer and Kazbek (alpha bitter acid contents varying from 2.5 to 8.0% and beta bitter acid contents varying from 2.0 to 6.0% in cones) and the “bitter” hop cultivars Vital, Herkules, Columbus, and Magnum (alpha bitter acid contents varying from 11.0 to 18.0% and beta bitter acid contents varying from 4.0 to 10.0% in cones) as contrasting hop genotypes for group analyses [2]. In 2020, three pooled samples of eight plants for each hop cultivar (Saaz, Fuggle, Hallertauer, Vital, Herkules, and Columbus) were collected from different tissues during development: apexes in April (only Saaz and Vital), flowers in July, leaves and young cones in August, and mature cones in August and September. Samples collected in August 2016 were used to increase the tissue variability. Three pooled samples containing one kilogram fresh weight of mature cones were used for bract and lupulin gland analyses in the hop cultivars Saaz, Fuggle, Hallertauer, Kazbek, Vital, Columbus and Magnum. Three pooled samples of four plants of the hop cultivars Saaz, Fuggle Columbus and Magnum collected in May 2013 were used to increase the variability of leaf and apex tissues. All samples were immediately frozen in liquid nitrogen and stored in a deep freezer (− 80 °C). Lupulin glands were separated from mature cones by agitation in liquid nitrogen followed by filtration through a 1 mm metal sieve to remove cone debris [1]. The glands were recovered from liquid nitrogen, and the rest of the cones were analysed as bracts without lupulin. We did not measure either the bitter acid contents or numbers of lupulin glands in hop cone samples.

### RNA isolation and gene expression analyses

RNA was isolated using PureLink™ Plant RNA Reagent (Thermo Fisher Scientific, Waltham, MA, USA) according to the manufacturer’s protocol and purified by DNaseI treatment on a column (RNeasy Plant Mini Kit, Qiagen, Hilden, FRG) [11]. RNA samples were reverse transcribed by oligo (dT)_18_ primer and a First Strand cDNA Synthesis Kit (Roche Diagnostics, Mannheim, Germany) at 50 °C for 60 min. NGS genome information [39] and the HopBase database [38, 40] were used to search for bitter acid biosynthesis [3] and trichome-specific transcription factor [1] gene sequences (Table 1). Genes were selected based on previous works [1, 3–14, 18–20]. Advanced BLAST 2.0 (http://www.ncbi.nlm.nih.gov/blast/blast.cgi) and the MegAlign module (LASERGENE system v. 7.1, DNAStar, Madison, WI, USA) were used to evaluate sequence homology. RealTimeDesign software (LGC Biosearch Technologies, Petaluma, CA, USA) was used to design real-time PCR primers (Table S1). A total of 2 μl of 50 × diluted cDNA was used in a 20 μl PCR with iTaq universal SYBR green Supermix (Bio–Rad Laboratories, Hercules, CA, USA) in a CFX Connect real-time PCR cycler (Bio–Rad Laboratories, Hercules, CA, USA). Five reference genes (Table 1), which were found to be constitutively expressed in different tissues [11] by NormFinder in GenEx v.6.0.1.612 (MultiD Analyzes AB, Gothenburg, Sweden), were used for normalization of the samples. The relative expression of five reference genes was calculated by the “delta-delta method” (RE = 2^-∆CT^) for each sample. SigmaPlot for Windows v.10.0.0.54 (Systat Software Inc., San Jose, CA) was used for statistical group (average, standard deviation) and unpaired two-sample t-test analyses of relative expression.

**Table 1:** List of analysed bitter acid biosynthesis gene, trichome-specific transcription factor and reference gene sequences

Abbrev.	Encoded proteins	Number^a^
	**Bitter acids biosynthesis**	
HlBCAT1	Branched-chain amino acid aminotransferase 1	002627F.g2 JQ063073
HlVPS	Phloroisovalerophenone synthase	001329F.g74 FJ554588
HlPT1L	2-Acylphloroglucinol 4-prenyltransferase	KM222441
HlPT2	2-Acyl-4-prenylphloroglucinol 6-prenyltransferase	KM222442
HlHS1	Monooxygenase 2 (humulone synthase 1)	010625F.g1 008956F.g7 KJ398144
HlHS2	Monooxygenase 2 (humulone synthase 2)	008118F.g14 KJ398145
	**Transcription regulation of bitter acid biosynthesis**	
HlMYB3	Transcription factor HlMYB3	AM501509
HlMYB7	Transcription factor HlMYB7	007341F.g6 FR873650
HlMYB8	Transcription factor HlMYB8	002031F.g25 HG983335
HlMYB78	Transcription factor MYB78	000063F.g63
HlbHLH2	Transcription factor HlbHLH2 (TT8)	000662F.g4 FR751553
HlWRKY1	WRKY transcription factor 1	000029F.g2 CBY88801
	**Transcription regulation of trichome development**	
HlETC1	Enhancer of TRY and CPC 1	GAAW01034579
HlCPC	MYB Transcription factor CPC (CAPRICE)	GAAW01028135
HlMYB61	Transcription factor MYB61	000756F.g12
HlMYB5	Transcription repressor MYB5	001020F.g25
HlMYB106	Transcription factor MYB106 (MIXTA)	000018F.g94
HlGL3	Transcription factor GLABRA 3 (HlbHLH4)	001145F.g21 HG983336
WRKY44	WRKY transcription factor 44	002199F.g12
HlGL2	Homeobox-leucine zipper protein GLABRA 2	002700F.g20
HlZHD6	Zinc-finger homeodomain protein 6	000360F.g51
HlHDG11	Homeobox-leucine zipper protein HDG11	004599F.g3
HlHDG2	Homeobox-leucine zipper protein HDG2	000903F.g16
HlPDF2	Homeobox-leucine zipper protein PROTODERMAL FACTOR 2	001505F.g11
HlHB14	Homeobox-leucine zipper protein ATHB-14	GAAW01071051
HlHB51	Homeobox-leucine zipper protein ATHB-51	005711F.g8
HlZFP4	Zinc finger protein 4	005456F.g11
HlZFP5	Zinc finger protein 5	000280F.g32
HlZFP6	Zinc finger protein 5	001196F.g44
HlZFP8	Zinc finger protein 5	001694F.g13
HlGIS3	Zinc finger protein GIS3	000092F.g31
HlYABBY1	Axial regulator YABBY 1	000464F.g10
HlRAV1	AP2/ERF and B3 domain-containing transcription factor RAV1	001017F.g6
HlABIL2	Abl interactor-like protein-2	000482F.g27
HlABIL3	Abl interactor-like protein-3	001806F.g14
HlCYCB2–4	Cyclin-B2–4	001015F.g12
HlCYCNB1	G2/mitotic-specific cyclin-1	006700F.g8
	**Reference genes**	
HlTTG1	Protein TRANSPARENT TESTA GLABRA 1 (HlWD40)	002162F.g15 FN689721
HlMYC2	Transcription factor MYC2	001862F.g5
HlPIF4	Transcription factor PIF4	000802F.g1
HlGAPDH	Glyceraldehyde-3-phosphate dehydrogenase	004935F.g2 004935F.g5
HlRH46	DEAD-box ATP-dependent RNA helicase 46	000004F.g75

^a^ HopBase gene number, NCBI GenBank Accession number or TSA number

## Supplementary Information

**Additional file 1.**

## Data Availability

The datasets used and/or analysed during the current study are available from the author on reasonable request.

## Abbreviations

*A. thaliana*: *Arabidopsis thaliana*
BCAA: Branched-chain amino acids
bHLH: Basic-helix-loop-helix
BLAST: Basic local alignment search tool
C2H2: Cys2His2
MBW: MYB-bHLH-WD40
MEP: Methylerythritol phosphate
MYB: Myeloblastosis
MYC: Myelocytomatosis
NGS: Next generation sequencing
qRT–PCR: Quantitative real-time polymerase chain reaction
RNA: Ribonucleic acid
WD: Tryptophan-aspartic acid dipeptide
